# Humoral immunoprofiling identifies novel biomarkers and an immune suppressive autoantibody phenotype at the site of disease in pancreatic ductal adenocarcinoma

**DOI:** 10.3389/fonc.2024.1330419

**Published:** 2024-02-21

**Authors:** Pamela Winnie M. Maimela, Muneerah Smith, Andrew J. M. Nel, Suba Dharshanan P. Bernam, Eduard G. Jonas, Jonathan M. Blackburn

**Affiliations:** ^1^ Department of Integrative Biomedical Sciences, Faculty of Health Sciences, University of Cape Town, Cape Town, South Africa; ^2^ Sengenics Corporation, Kuala Lumpur, Malaysia; ^3^ Department of Surgery, Gastroenterology Unit, Groote Schuur Hospital, University of Cape Town, Cape Town, South Africa; ^4^ Institute of Infectious Disease and Molecular Medicine, Faculty of Health Sciences, University of Cape Town, Cape Town, South Africa

**Keywords:** protein microarray, autoantibody biomarkers, antibody glycosylation, cancer testis antigen, pancreatic ductal adenocarcinoma

## Abstract

Pancreatic ductal adenocarcinoma (PDAC) is a heterogeneous cancer, with minimal response to therapeutic intervention and with 85% of cases diagnosed at an advanced stage due to lack of early symptoms, highlighting the importance of understanding PDAC immunology in greater detail. Here, we applied an immunoproteomic approach to investigate autoantibody responses against cancer-testis and tumor-associated antigens in PDAC using a high-throughput multiplexed protein microarray platform, comparing humoral immune responses in serum and at the site of disease in order to shed new light on immune responses in the tumor microenvironment. We simultaneously quantified serum or tissue IgG and IgA antibody isotypes and subclasses in a cohort of PDAC, disease control and healthy patients, observing inter alia that subclass utilization in tumor tissue samples was predominantly immune suppressive IgG4 and inflammatory IgA2, contrasting with predominant IgG3 and IgA1 subclass utilization in matched sera and implying local autoantibody production at the site of disease in an immune-tolerant environment. By comparison, serum autoantibody subclass profiling for the disease controls identified IgG4, IgG1, and IgA1 as the abundant subclasses. Combinatorial analysis of serum autoantibody responses identified panels of candidate biomarkers. The top IgG panel included ACVR2B, GAGE1, LEMD1, MAGEB1 and PAGE1 (sensitivity, specificity and AUC values of 0.933, 0.767 and 0.906). Conversely, the top IgA panel included AURKA, GAGE1, MAGEA10, PLEKHA5 and XAGE3aV1 (sensitivity, specificity, and AUC values of 1.000, 0.800, and 0.954). Assessment of antigen-specific serum autoantibody glycoforms revealed abundant sialylation on IgA in PDAC, consistent with an immune suppressive IgA response to disease.

## 1 Introduction

Pancreatic ductal adenocarcinoma (PDAC) is a profoundly heterogeneous disease with a highly diverse molecular architecture that affects the immune response elicited against the tumor (1). Moreover, the anti-tumor response of immune cells depends on their functional phenotype, cellular composition, and autoantigen specificity and abundance. The overall survival rate of pancreatic cancer is reported to be less than 5% (2). This is attributed to its difficult diagnosis and poor prognosis. Indeed, most cases are diagnosed at a late stage, where curative surgery is almost impossible, and despite advances in cancer therapy, pancreatic cancer remains difficult to treat due to a lack of druggable targets. Furthermore, most patients with pancreatic cancer fail to display symptoms at an early stage, and the identified symptoms are associated with other abdominal diseases such as dyspepsia and pancreatitis.

Similar to autoimmune disorders, cancer produces autoantibodies. Typically, these autoantibodies are raised against mutated or aberrantly expressed/modified proteins. Thus, autoantibody-based tumor biomarkers have been studied as potential prognostic, diagnostic, and monitoring agents of therapeutic response in breast (3, 4), prostate (5, 6), and lung (7, 8) cancers amongst others. The common challenge with these biomarker studies is that the identified targets individually typically lack specificity and sensitivity, and some are only applicable to a specific tumor subset. As a result, the clinical application of antibodies in cancer to date has largely focused on their use as targeted immunotherapies. Of note, there are five human antibody isotypes, yet current therapeutics are based on the IgG isotype, particularly IgG1 (9), with other antibody isotypes having not been explored or developed for mAb therapies. Similarly, the aforementioned studies on autoantibody-based biomarkers were based solely on detection of IgG antibodies, yet mucosal involvement in solid tumors makes it likely that there will also be significant, yet-to-be-discovered IgA responses in such cancers. Notably, the literature has shown that IgA antibodies have effector functions that can be targeted for biomarker applications in therapeutic development. Indeed, a study by Brandsma et al. compared Fc receptor signaling between IgG and IgA and reported that IgA mediated higher tumor lysis with neutrophils (10). Furthermore, Steffen et al. demonstrated that the effector function and binding of IgA subclasses to neutrophils and macrophages were influenced by their glycosylation profiles (11). Moreover, IgA complexes are key regulators of the immunopathogenesis of IgA nephropathy and vasculitis (12). Currently, no single antigen has been used alone as a cancer biomarker because of the low sensitivity and specificity of single markers. However, biomarker panels consisting of multiple tumor antigens can, in principle, result in high sensitivity and specificity. Therefore, multiplexed analysis of cancer biomarker targets using autoantibodies to detect specific cancer types, even at early stages, can address the unmet clinical need for biomarker development. Additionally, literature has shown that plasma and B cells are present in the tumor microenvironment and that the cognate antibodies they produce can drive a specific immune response (13, 14). In the tumor environment, B cells can present cancer antigens to facilitate T-cell responses such as the release of cytokines as an anti-tumor response (1, 15). Additionally, they can produce specific antibodies targeted against tumor antigens at the site of disease (13). In turn, the antibody repertoire would produce distinct immune functionalities that would either be immunosuppressive or immunocompetent (1). Since cancer antigens are produced *de novo* at the site of disease (16), determining the local autoantibody response and identifying specific antibodies driving the immune response against these antigens should aid in tumor characterization, whilst also increasing understanding of the dichotomy associated with cancer immunity.

A major advantage of using autoantibodies for cancer diagnosis is that increased autoantibody levels can be detected at early stages of the disease, and their production may occur months to years before any clinical signs of tumor development are presented (17). In searching for novel autoantibody biomarkers in cancers, the target search space is a critical consideration, since the theoretical human proteome is vast, at >1m proteoforms, yet not all proteins have an equal propensity to be mis-recognized an autoantigen in disease (18). Tumor antigens can be defined as tumor associated (TA) antigens, which are antigens similar to proteins found in normal cells but are modified or aberrantly expressed. Moreover, amongst the subset of proteins that are more likely to elicit an autoantibody response in cancers, the cancer-testis (CT) antigens are a family of ca. 500 tumor-specific antigens with highly restricted expression in normal adult somatic tissues and aberrant expression in various cancers as a result of disrupted gene regulation (19–22). As the testis is an immune-privileged site, aberrant expression of these antigens in cancer typically triggers a spontaneous cellular (T cell) and humoral (B cell) immune response to the relevant CT antigen (23, 24). The latter includes the maturation of B cells against specific antigens to produce cognate antibodies which are detectable in the circulation (25). Notably, assay of >3000 healthy individuals showed no detectable anti-CT antigen autoantibody titer [Duarte and Blackburn, unpublished]. Autoantibodies against CT antigens thus represent particularly attractive cancer diagnostic targets, due to this highly cancer-specific signal which derives from the site of disease but is measurable in the periphery.

However, it is also important to remember that antibody production is compartmentalized, with bone marrow-derived B-cells and tissue resident B-cells having distinct lineages and producing different antibody isotypes, potentially against different target antigens, which can confuse interpretation of the physiological significance of autoantibody production in cancers. Here, we therefore used a high-throughput multiplex microarray platform to profile serum samples for autoantibody responses, quantifying CT and TA antigen-specific IgG and IgA isotypes and their respective subclasses in PDAC and comparing them to autoantibodies extracted from diseased tissue. Moreover, because different subclasses of each antibody isotype have varying effector functions, which are also dependent on the glycan composition of the Fc region, their characterization would better reflect the antibody effector roles that are responsible for immune regulation in PDAC carcinogenesis. Thus, we aimed to identify the glycan moieties associated with the antigen-specific autoantibodies identified in serum. Through this study, we quantify the differential autoantibody responses found at the site of disease compared to the periphery and provide new insights regarding local autoantibody production in an immune tolerant tumor microenvironment in PDAC.

## 2 Materials and methods

### 2.1 Sample collection for study cohort

This study was approved (HREC 654/2017 & HREC 802/2020) by the Human Research Ethics Committee of the Faculty of Health Sciences, University of Cape Town. Blood samples were obtained from patients with early-stage (stage 1A, 1B or 2A) pancreatic ductal adenocarcinoma (n=30) who were diagnosed and underwent tumor resection surgery (pancreaticoduodenectomy) at Groote Schuur Hospital (GSH), Cape Town, South Africa. Informed consent was obtained from all patients involved in the study. Additionally, serum from patients with confounding diseases of the pancreas, chronic pancreatitis (n=16), non-ulcer dyspepsia (n=13) were used as the disease controls, and from patients characterized as having a latent tuberculosis infection (LTBI) (n=30) but who were otherwise healthy, were used as ‘healthy’ controls. Notably, in a South African context, public health data suggests that ca. 80% of adults carry a latent tuberculosis infection, thus making the LTBI group an appropriate control in the present study.

Tissue matched from a subset of PDAC patients in the serum cohort was collected for assessment of local autoantibody production. Here, paired tissue sections of ~3mm^2^ for PDAC tumour (n=8), normal adjacent (n=8), and chronic pancreatitis (n=8) were lysed for antibody extraction as previously described (26) and antibody presence in tissue lysates was confirmed by an immunoglobulin affinity purification method using magnetic Protein A and Protein G microbeads (MagReSyn®), as per to the manufacturer’s protocol, and as previously described (27, 28) Serum and tissue samples were stored at -80 until assays were performed.

#### 2.1.1 Serum and tissue antibody assays

Antibody assays were performed as previously described (29), with the following modifications: A dual colour format was adapted for antibody detection with simultaneous incubation of AF647-labelled anti-human IgG, and AF555-labelled anti-human IgA detection antibody (both at 10µg/ml) for 30 min. Similarly, dual-colour microarray assays were performed for the respective antibody subclasses. The serum autoantibody and lectin microarray assays were carried out using commercial cancer-testis antigen arrays comprising 213 CT plus 49 TA antigens (Sengenics), whereas microarray assays on tissue extracts were performed on a custom CT100^+^ platform as previously described (30). Antigen content for both of these array platforms is provided in **Supplementary Table S1**. Both arrays contained triplicate spots of each antigen, and individual arrays were isolated using ProPlate 4-plex multi-well chambers. Detection antibody subclasses (anti-human IgG1, IgG2, IgG3, IgG4, IgA1, and IgA2) and anti-human IgA were derivatized in-house with either Alexa Fluor (AF)647 or AF555 (**Supplementary Table S2**). The assays were performed with a serum or tissue lysate dilution of 1:200 in phosphate-buffered saline with Tween20 (PBST), with minimal light, at room temperature, and all incubations were performed on a shaker at 100 rpm unless stated otherwise.

#### 2.1.2 Serological lectin assays

Microarray slides were washed with PBST and incubated with gentle agitation for 3x 5 min, then washed with 2x 5min with PBS and dried by centrifugation at 1200*X* g for 2min. Individual arrays were incubated with the patient serum for 1hr with gentle agitation. Thereafter, the slides were briefly rinsed 3x with Tris-buffer saline (TBS) and incubated with 3% deglycosylated BSA for 1hr. Subsequently, each lectin was diluted to 1ug/ml in lectin binding buffer (20 mM Tris (pH 8.0), 0.1 mM CaCl_2_, 0.1 mM MgCl_2_, 0.1m M MnCl_2_, 0.2% Tween 20), added to the slide, and incubated with gentle agitation for 30 min. Finally, the slides were washed 2x for 5 min each with TBST and TBS.

### 2.2 Bioinformatic analysis

The statistical estimation of power and sample size were calculated using power calculations and performed using G*Power version 3.1.9.4. *Post hoc* power calculations of matched tumor and normal-adjacent tissue was performed using the R package “ssize.fdr” (31–33).

#### 2.2.1 Microarray image analysis and raw data extraction

Microarray slides were scanned at a fixed gain setting using an InnoScan 710 (Innopsys, Carbonne, France) fluorescence microarray scanner, generating a 16-bit TIFF file. A visual quality control check was conducted and any arrays showing artifacts were re-assayed. A GAL (GenePix Array List) file containing information regarding the location and identity of all antigen spots was used for image analysis. Automatic extraction and quantification of each spot were performed using Mapix software (Innopsys) to obtain the median foreground and local background pixel intensities for each spot.

#### 2.2.2 Data pre-processing and statistical analysis

The mean net fluorescence intensity of each spot was calculated as the difference between the raw mean pixel intensity and its local background using in-house developed software [Protein Microarray Analyser (34);]. The output files contained the relative fluorescent unit (RFU) and coefficient of variation for all antigens and controls spotted on the array. The R studio and R packages were used to perform clustering analysis, and the OptimalCutPoints package and receiver operating curves (ROC) were used to determine antigen specificity and sensitivity. Combinatorial ROC analysis (http://combiroc.eu/) was used to determine the potential antigen biomarker panels. Other statistical analyses and graphical representations were generated using GraphPad Prism (v 9.5.1; GraphPad Software, San Diego, CA, USA).

## 3 Results

### 3.1 Quantifying autoantibody responses against cancer testis antigens

To determine the specific autoantibody reactivity against the cancer antigens on our microarray platforms, we set a signal intensity threshold to distinguish true antibody-antigen binding from non-specific binding. After data normalization, the threshold for each protein was set as the mean plus 2SD for the normal-adjacent tissue samples (for tissue extracts) or the control group for serum; proteins with signals above this threshold were considered true autoantibody binding.

It is well understood from ligand binding theory that the relative fluorescent units (RFU) measured for autoantibodies bound to individual autoantigens on a protein microarray depends on the density of the immobilized autoantigen, the concentration of the autoantibody in solution and the affinity of interaction between the autoantigen and autoantibody. Furthermore, the fluorophore labelling efficiency will vary between different isotype-specific detecting antibodies. Thus, whilst it is meaningful to compare RFU values for a given isotype bound to the same autoantigen across different samples, it is generally not considered meaningful to directly compare RFU values between the same isotype bound to different autoantigens on a microarray, or between different isotypes bound to the same autoantigen. However, RFU values for each autoantigen-bound autoantibody are nonetheless linearly related to antibody concentrations (titers).

**Figure 1:**
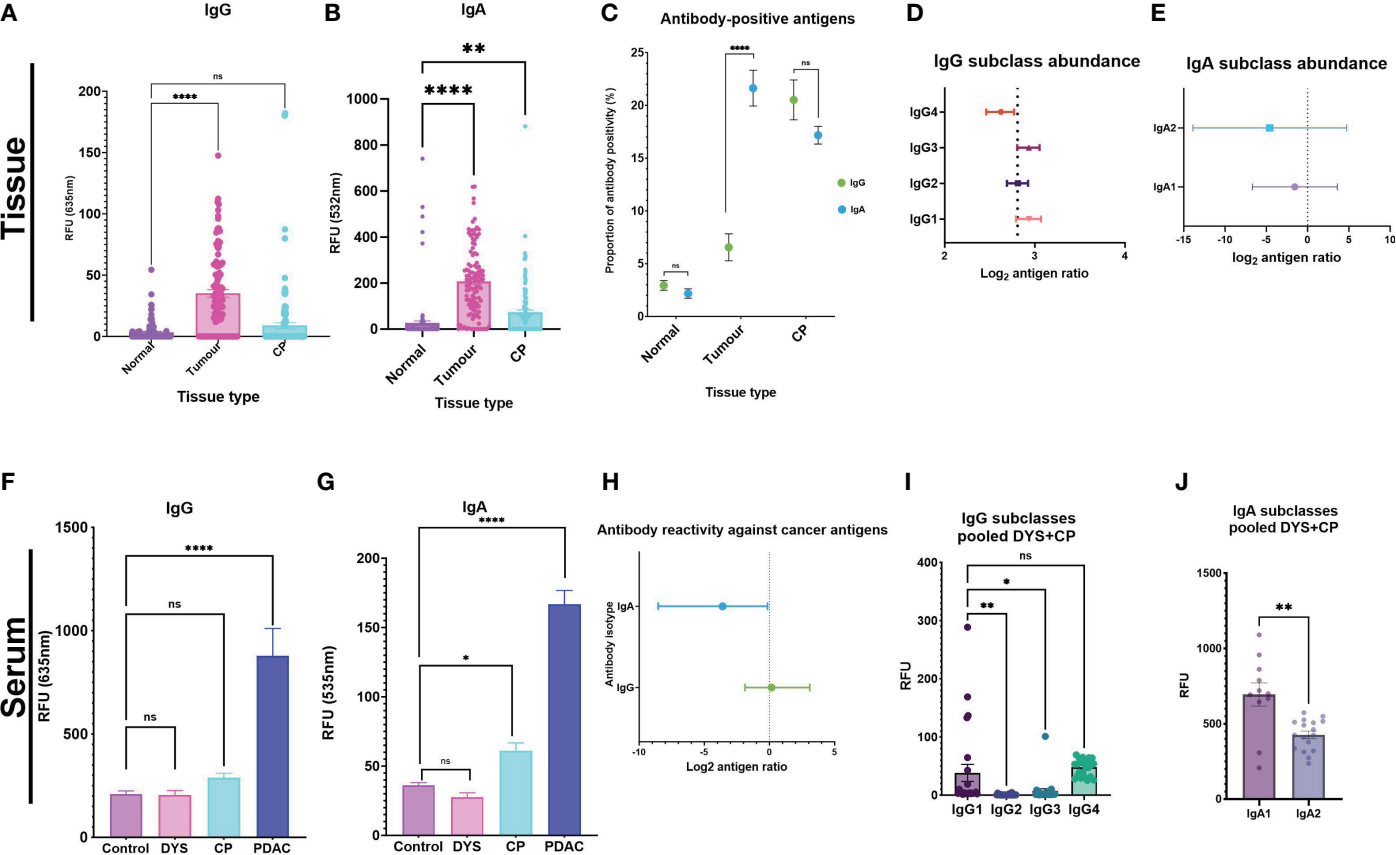
Autoantibody response against tumor antigens in PDAC serum and tissue samples. Tissue analysis for **(A)** IgG and **(B)** IgA were used as surrogate indicators of antibody-isotype abundance between the three tissue types. **(C)** The proportion of antibody-positive antigens was also compared for IgG and IgA across the three tissue types. Forest plots showing the antibody subclass abundance for IgG and IgA antibody-isotypes in tissue. **(D)** Antibody subclass abundance determined by calculating the mean difference between antigen ratios (mean isotype antigen ratio – mean subclass antigen ratio), and the 95% and 5% confidence interval (CI) and represented on a forest plot. The four IgG subclasses show IgG4 as the predominant antibody response in PDAC tissue, IgG1 and IgG3 have similar abundance. **(E)** IgA2 is the predominant IgA subclass, and IgA1 is comparably elevated in PDAC tumor tissue. Serum analysis showing bar graphs for differences in signal intensity values between cohorts with the pooled DYS+CP cohort segregated into two groups of Dyspepsia (DYS) and chronic pancreatitis (CP) for IgG and IgA. **(F)** IgG response shows that both confounding groups (CP and DYS) have no significant difference to the control group. **(G)** The CP group has a significantly different response to the control group for IgA, while the DYS group has a similar response to the control group. **(H)** Forest plot comparing abundant isotype distribution of antibody-positive antigens, showing that more antigens are IgA-positive. **(I)** Column graph showing the distribution of IgG subclasses based on signal RFU in the pooled DYS+CP cohort. **(J)** IgA subclass reactivity for the pooled DYS+CP group, indicating abundant IgA1 compared to IgA2. Signal intensity values for normal-adjacent, tumor, and chronic pancreatitis (CP) tissue, and serum samples for PDAC, CP,DYS, and LTBI were set at a threshold > mean+2SD of the normal-adjacent antibody-positive antigen response and the scatter plots showing the relative fluorescent units (RFU) of the mean ± SEM for Statistical analysis by one-way ANOVA, pairwise comparison of each group against the control group, graph represents mean values SEM (*P ≤ 0.05, **P ≤ 0.01, ***P ≤ 0.001, ****P ≤ 0.0001); ns, not significant.

Given moreover that, when comparing autoantibody profiles in PDAC *vs* chronic pancreatitis (CP) and other controls, in serum and in tumor or normal-adjacent samples, different autoantigens were identified in the different disease and sample types, we therefore plotted all antigen-specific RFU values for each autoantibody isotype (IgG; or IgA) in each sample type (tumor-; CP-; or normal-adjacent tissue) in order to provide a measure of relative autoantibody isotype abundance (**Figures 1A, B**). The assumption being made here is that an increased number of autoantibody-positive antigens for a specific isotype (**Figure 1C**) should result in an increased mean autoantibody RFU value for that isotype. Thus, **Figures 1A–C** should be read together since they provide two different dimensions of relative autoantibody isotype abundance: number of autoantigens per isotype and mean titers of antigen-specific autoantibodies per isotype.

For the tissue samples, *post-hoc* power calculations indicate that a single comparison of the paired PDAC tumor (n=8) and normal-adjacent (n=8) provides >99% power to detect a 5-fold change in IgG/IgA ratios either direction (FDR 0.05). Thus, the microarray results show that whilst there was little difference observed for the IgG levels between CP tissue and normal-adjacent tissue, significant differences were found in IgG levels between normal-adjacent and tumor tissue (p<0.0001) (**Figure 1A**). The autoantibody levels for IgA also showed a significant difference for both tumor and CP tissue compared to the normal-adjacent samples (**Figure 1B**). Furthermore, IgA had a higher RFU compared to IgG in the tissue samples, with a 1:5 IgG/IgA ratio. These results thus provide an estimation of the abundant autoantibody isotype in pancreatic tissue and indicate an IgA-dominant cancer tissue microenvironment. In accordance with autoantibody abundance, the proportion of autoantibody-positive antigens showed significantly higher (p<0.0001) levels of IgA-positive antigens in tumor samples compared to IgG-positive antigens, but no statistically significant difference was observed for the normal-adjacent and the CP tissue samples (**Figure 1C**). Together, these results indicate that in tissue, IgA-based responses against cancer antigens predominate in PDAC, but isotype-based specificity is not established in CP. Furthermore, we investigated the abundance of the respective subclasses for each antibody isotype, by calculating the antigen ratio (number of antibody-positive antigens number of antibody-negative antigens) for each subclass and corresponding isotype in each sample and then representing the mean difference between the two for each sample type (mean isotype antigen ratio – mean subclass antigen ratio), together with the 95% and 5% confidence interval (CI), for each subclass in a forest plot, in order to enable comparisons to be made independent of differences in isotype abundance between patients. The smaller and likely more negative the resultant mean difference, the greater the abundance of the subclass within the respective isotype. In tissue, the majority of IgG antigen-bound autoantibodies were found to be of the IgG4 subclass (**Figure 1D**). Moreover, we observed that the complement activating subclasses, IgG1 and IgG3, were the least abundant in the tumor environment of PDAC. The IgG subclass data thus indicated a tolerogenic immune response because the dominant antibody, IgG4, is considered a non-activating antibody. Interestingly, IgA subclass autoantibodies targeting tumor antigens in PDAC had a similar abundance, but with higher IgA2 than IgA1, as expected (**Figure 1E**). Although IgA1 and IgA2 mediate their effector functions by binding to the FcαRI as monomers, they are considered to be regulatory and pro-inflammatory respectively, with IgA2 typically dominant in mucosal tissue. Moreover, in the tissue extracts, it is likely that we are detecting dimeric sIgA, which is produced by mucosal plasma cells, rather than monomeric IgA (produced by bone marrow-derived B-cells). Together, the antibody subclass data from tissue samples indicates an antibody response that is pro-inflammatory, and not optimal for the activation of effector functions, thus promoting a tolerogenic tumor environment in PDAC.

By contrast, when we quantified the mean signal intensities of IgG and IgA in serum, we observed that the overall mean intensity values were ~5-fold higher for IgG (**Figure 1F**) than for IgA (**Figure 1G**), as expected. Thereafter, we assessed the abundance of autoantibody isotypes between IgG and IgA by calculating the antigen ratio for each isotype (number of antibody-positive antigens number of antibody-negative antigens), and the 95% and 5% confidence interval (CI) and represented these ratios as a forest plot. The results showed that IgA had a high abundance of positive anti-CT/TA antigen autoantibody signals compared to IgG (**Figure 1H**), albeit IgG had a higher RFU signal overall.

Furthermore, serum antibody subclass profiles - obtained by enumerating the number of antigens that had a positive response for a specific subclass in each patient - indicated that the most abundant isotypes in serum were IgG3 and IgG4, followed by IgG1, and the least abundant isotype was IgG2. Generally, in serum the overall pattern for all the patients indicated a dominant IgA subclass reactivity against the cancer antigens, with variation on the preferred subclass, indicating the significance of an IgA directed anti-CT/TA antigen response in PDAC. Interestingly, we observed a reactivity pattern that showed that patients (n=4/30; 13%) who had a dominant IgA subclass reactivity that was strong or very strong for both subclasses, had no IgG1 reactivity. The serum autoantibody subclass profiling of each patient is summarized in **Table 1**. By contrast, the serum autoantibody subclass profiling for the pooled DYS+CP group indicated IgG1, IgG4 and IgA1 as the abundant subclasses (**Figures 1I, J**).

**Table 1:** A summary of individual autoantibody subclass reactivity in serum outlining the presence and frequency of antibody-positive antigen frequency per patient.

Patient (Px) ID	Antibody subclass					
	IgG1	IgG2	IgG3	IgG4	IgA1	IgA2
**Px1**	+	+++	++	++	+++	+
**Px2**	–	+	++	++	++	++
**Px3**	–	+	++	++	+++	+++
**Px4**	–	+	++	++	+++	++
**Px5**	+	+	++	+++	++	+
**Px6**	+	–	++	++	+++	+++
**Px7**	+	–	++	++	++++	+
**Px8**	+	+	++	++	PSA	++
**Px9**	+	+	+++	++	++	+++
**Px10**	++++	+	++	++	++	+
**Px11**	+	–	++	++	+++	+
**P12**	+	+	++	++	PSA	++
**P13**	+	+++	+++	++	PSA	+
**Px14**	+	+	++	++	++	+
**Px15**	+	+	++	++	PSA	+
**Px16**	–	+	++	++	++++	+++
**Px17**	+	+	++	+++	+	++
**Px18**	+	+	+++	++	++++	+
**Px19**	–	+	+	++	++++	++
**Px20**	+	+++	++	++	PSA	+
**Px21**	+	++	++++	++	++++	++
**Px22**	+	+	++	++	++	+
**Px23**	–	+	++	++	PSA	++
**Px24**	+	+++	++	++	++++	+
**Px25**	–	+	++	+++	+++	PSA
**Px26**	+	+++	++	++	+++	+
**Px27**	+	+++	PSA	++	+++	+
**Px28**	–	++	++	++	PSA	PSA
**Px29**	+	+	+	++	++	+
**Px30**	++	–	++	++	+++	++

Symbol key: PSA (Polyspecific antibody “sticky phenotype” i.e., antigen frequency > n=80) **++++** (very strong; n= 64-80) **+++**(strong; n=48-63) **++** (moderate; n=30-47) + (weak; n=1-29) **–** (absent; n=0).

### 3.2 Identification of candidate autoantibody-based serum biomarkers

The minimal invasive nature of acquiring liquid biopsies makes them attractive candidates for cancer diagnosis, monitoring, and characterization. Thus, we aimed to identify the most significant cancer antigen-specific autoantibodies from our study and to determine a subset of those autoantibodies that could be used as a panel of candidate serum biomarkers. Here, we obtained 15% of IgG-positive significant antigens, compared to 61% for IgA-positive antigens, and 24% of the identified significant antigens were shared between the two antibody isotypes. Thereafter, we applied a false discovery rate (FDR) of 1% using the Benjamini-Hochberg method (35), after which no IgG-reactive antigens were retained individually as candidates. However, there were 12 IgA-reactive antigens retained as candidates after applying a 1% FDR. Subsequently, we performed multiplex analysis of our data by combinatorial ROC analysis on all antigens that were identified as being significantly different, with comparisons between the PDAC group and the pooled DYS+CP group. This allowed us to determine a subset of antigen combinations with the best specificity and sensitivity as potential PDAC biomarkers, thus creating a panel of antigens instead of single biomarkers. The top five antigen combinations for IgG and IgA are summarized in **Table 2**. The top IgG combinations had AUC, sensitivity, and specificity values of 0.906, 0.933, and 0.767, respectively. The top IgA combination showed AUC, sensitivity, and specificity values of 0.968, 1.00, and 0.833, respectively.

**Table 2:** Combinatorial ROC analysis of top 5 antigen combinations for serum IgG and IgA PDAC classifiers showing the area under the curve (AUC), Sensitivity, and specificity values for each antigen combination.

Autoantibody	Combination symbol	Antigens	AUC	sensitivity	specificity
*IgG*	I	GAGE1-LEMD1-MAGEB1-PAGE1	0.824	0.800	0.767
	III	GAGE1-LEMD1-MAGEB1-TSGA10	0.833	0.833	0.800
	XXIX	ACVR2B-BAGE4-GAGE1-LEMD1-MAGEB1-TSGA10	0.899	0.800	0.800
	VII	ACVR2B-GAGE1- LEMD1-MAGEB1-TSGA10	0.901	0.900	0.733
	VI	ACVR2B-GAGE1- LEMD1-MAGEB1-PAGE1	0.906	0.933	0.767
*IgA*	X	AURKA-GAGE1-MAGEA10-MAGEB1	0.956	1.000	0.833
	XXIX	AURKA -MAGEA10-MAGEB1-PAGE1	0.940	0.900	0.867
	XXX	AURKA -MAGEA10-MAGEB1-PLEKHA5	0.968	1.000	0.833
	LXXV	AURKA -GAGE1-MAGEA10-MAGEB1-PLK4	0.963	0.967	0.933
	LXXVII	AURKA -GAGE1-MAGEA10-PLEKHA5-XAGE3Av1	0.954	1.000	0.800

### 3.3 Antigen-specific autoantibody glycosylation patterns differ between PDAC and confounding cohort

**Figure 2:**
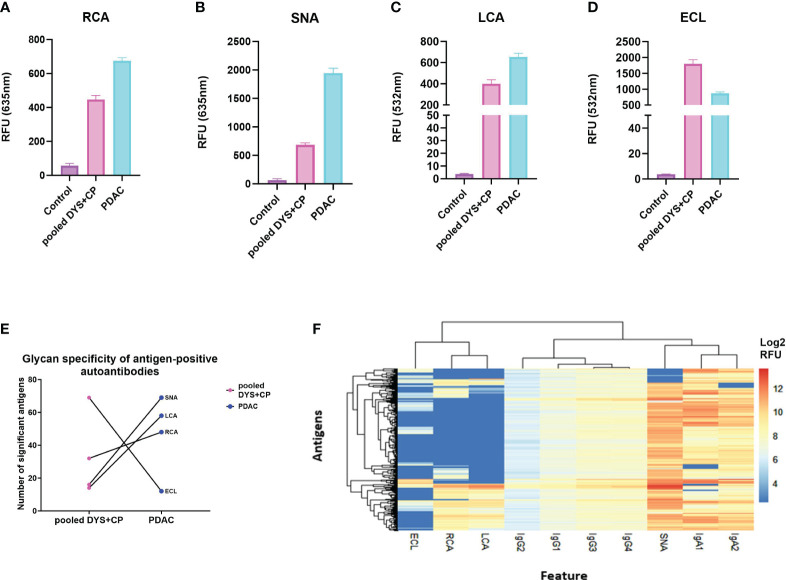
Comparing glycosylation features of serum autoantibodies against CTAs in PDAC. **(A)** The total glycosylation distribution for the PDAC, pooled DYS+CP, and control cohort for galactosylation, fucosyaltion, and sialylation. Column graphs showing the mean SEM relative fluorescent units (RFU) of antigen-bound autoantibodies (threshold set mean+2SD of the control RFU) that have glycans recognized by **(A)** RCA (β1,4-Gal), **(B)** SNA (α2,6-Sal), **(C)** LCA(α1,6-Fuc), and **(D)** ECL(β1,4-Gal) as a surrogate marker for assessing the detection of glycosylation moieties in serum autoantibodies using the Sengenics CT262 microarray platform. **(E)** Glycosylation features assessed in lectin-positive antigens observed in the PDAC cohort compared to the pooled DYS+CP group, showing the variable presence of different carbohydrate moieties. **(F)** A heatmap and dendogram of log_2_ transformed median normalized microarray data for each antigen-positive serum autoantibody feature and the associated detection of cancer testis antigens. Hierarchical clustering has grouped serum features and positive antigens by similarity.

Having confirmed autoantibody reactivity against specific cancer-testis antigens on our microarray platform, we aimed to determine whether there were differential glycan moieties on the antigen-specific autoantibodies in PDAC and controls. We achieved this by adapting a fluorescently-labelled lectin-based protocol from published bead-based assays for detecting antibody glycosylation patterns (36), profiling the glycoforms present on antigen-bound autoantibodies, detecting with 1µg/ml of RCA (β1,4-Gal), ECL(β1,4-Gal), SNA (α2,6-SA), or LCA(α1,6-Fuc), for the presence of each respective glycoform. As above, we set a threshold and defined a true positive signal as any relative fluorescent unit (RFU) that was above the mean+2SD of the control RFU. Thereafter, the mean standard error of the mean (SEM) for all the positive antigens was used as a measure of the total galactosylation, sialylation, and fucosylation levels in each cohort. The investigated glycoforms had uniform low signal intensities across all the lectin-specific glycans for the control group (**Figures 2A–D**), further validating the specificity of the assay on a cancer antigen microarray platform. Moreover, the data shows relatively high sialylation (**Figure 2B**), and fucosylation (**Figure 2C**) for the PDAC cohort compared to the pooled DYS+CP cohort. These results suggest that although the antigen-bound autoantibodies in the pooled DYS+CP cohort may be sialylated and fucosylated, the carbohydrate content varies between the two disease conditions, favoring increased α2,6-Sal and α1,6-Fuc in PDAC. However, the galactosylation levels show contrasting outcomes respectively showing high (**Figure 2A**) and low (**Figure 2D**) galactosylation for PDAC compared to the pooled DYS+CP group. Although both RCA and ECL have selectivity for β1,4-Gal, their respective binding affinity is impacted by the presence of other carbohydrate groups, particularly sialylation for ECL (37), suggesting that the glycosylation features between PDAC and pooled DYS+CP diseases of the pancreas are notably distinct. Furthermore, we assessed the relationship between the observed glycan motifs between PDAC and the pooled DYS+CP group. Significance testing was followed by pairwise testing, adjusting the antigen significance level based on probability value (*p-value*) rank order by employing the stringent Bonferroni correction to limit the risk of a type I error from multiple pairwise tests performed on the data. Subsequently, we plotted each disease condition against the corrected significant antigen count for each lectin (**Figure 2E**). The results show a marginal increase for RCA binding (*p-value* =0.553), and a significant increase for SNA (*p-value <*0.0001) and LCA binding (*p-value*<0.0001) for PDAC. In agreement with the above galactosylation data, we observed a significant (*p-value <*0.0001) decrease in the ECL (β1,4-Gal) motif in our PDAC cohort. Therefore, there are differences in the carbohydrate composition and motifs of serum autoantibodies identified for PDAC and pooled DYS+CP diseases of the pancreas, and these may be indicative of differential Fc region glycosylation that exists between serum autoantibodies present between the two different disease states. Thereafter, based on the lectin assay data of glycan-positive autoantibodies, and the data generated from autoantibody subclass assays, we performed unsupervised hierarchical clustering to determine whether the serum autoantibody signatures of the PDAC cohort could be associated with the observed glycosylation features (**Figure 2F**). To ensure comparability and avoid clustering driven by technical variation between signals for the same autoantigen in different samples, the log2 transformed data was scaled to the standard deviation. Antibody and lectin features form distinct clusters, with IgA subclasses showing shared characteristic antigen binding with the SNA (α2,6-Sal) lectin, indicating that both interactions are similar, and represent the serum autoantibody profile of our PDAC cohort. This data thus suggests that increased sialylation in both IgA1 and IgA2 is an immune feature of PDAC.

## 4 Discussion

Numerous studies have identified the presence of autoantibodies in cancer (4, 8), but the immunological importance of those antibodies in terms of whether they contribute to, or hamper cancer progression remains a matter of on-going debate. Part of the complication is that autoantibodies are typically detected in serum or plasma, making it unclear whether the measured signals derive from antibody production at the site of disease, or in the periphery, yet these two possibilities are not equivalent: in mucosal tissues, IgA is generally considered the dominant antibody isotype, whereas in blood, IgG dominates; moreover, tissue-resident B-cells in mucosa are of a different lineage to bone marrow-derived B-cells and primarily produce dimeric, secretory IgA, whereas IgA in blood is primarily monomeric IgA1. Antigen specificity can in principle also vary between blood and mucosal tissues due to the different B-cell type at the site of production. Furthermore, it is well understood that (auto)antibody function is controlled by the specific isotype, subclass and glycosylation patterns, with for example IgG1 considered to be tumoricidal whereas IgG4 is considered immune suppressive (38). It is thus perhaps not surprising that measuring only IgG titers in blood has yielded conflicting results to date, since it lacks information concerning these nuances.

In order to provide more detailed characterization of the humoral response at the site of disease in PDAC patients, we therefore carried out quantitative autoantibody profiling against 262 cancer-testis and tumor-associated antigens, utilizing a multiplexed, reproducible high through-put microarray platform to determine the isotype, subclass, and sialylation of antigen-specific autoantibodies in serum and matched tumor tissue from PDAC patients and controls.

Amongst others, our data reveals significant differences in anti-CT/TA antigen IgG and IgA autoantibody titers between PDAC patients and controls that are measurable in serum and which, if the optimal autoantibody panels validate in an independent cohort, may provide the basis for early detection of PDAC; that validation is the subject of on-going research and will be reported elsewhere.

Interestingly, our data also reveals significant differences in anti-CT/TA antigen isotype and subclass utilization in tissue biopsies at the site of disease (predominantly IgA2 and IgG4) compared to that found in matched sera (predominantly IgG3 and IgA1) or in matched adjacent normal biopsies, which argues against simple infiltration of antibodies from blood into the diseased tissue and instead argues for local autoantibody production at the site of disease.

To our knowledge, the present study is thus the first to use a functional protein microarray platform to assess antigen-specific autoantibody responses at the site of disease in cancers, at a subclass level. In particular, functional antibody repertoire profiling of tissue extracts provides a novel means to quantitatively readout many features that report on the functional immune environment in tumors. This is relevant in characterizing the immune infiltrate and the associated immune regulation because differences in these features can reflect differences in immune responses responsible for disease onset and progression (15, 39). Through this approach, we have shown that the identified anti-CT/TA antigen autoantibody responses predominantly reflect a tolerogenic antibody response at the site of disease: The predominant anti-CT/TA antigen IgG subclass was found to be the non-activating IgG4, whereas IgG1 and IgG3 – which can induce antibody-dependent cellular cytotoxicity (ADCC) and complement dependent cytotoxicity (CDC) through their effector functions - were found to be the least abundant IgG subclasses in tissue, implying that these functions are not effectively activated to drive tumor clearance; and the predominant anti-CT/TA antigen IgA subclass was found to be the pro-inflammatory IgA2.

Antibodies are produced in the adaptive phase of an immune response and increased affinity for antigens is achieved through somatic hypermutations and isotype switching. Previously, studies have largely focused on IgG profiling in cancer and have demonstrated elevated expression levels of IgG in cancer cells (40, 41). By simultaneously measuring IgG and IgA responses in PDAC, here we have shown that whilst signal intensities were higher for IgG compared to IgA in serum, IgA has a broader selectivity for CT/TA antigens compared to IgG in both serum and tissue. Isotype switching increases the functional diversity of antibodies as the immune response proceeds. Indeed, the type of antibody activated during an immune response is important in determining downstream effector signaling and interaction with other immune cells. Previous studies have demonstrated that antibody isotype and subclass abundance fluctuate through the course of infectious diseases (42, 43) and it is plausible therefore that the same phenomenon occurs in cancers. Whilst the cohort studied here was cross-sectional, not longitudinal, in design, it is nonetheless interesting that in our serum analysis, patient IgG subclass evaluation revealed that IgG1 and IgG2 reactivity was absent in some patients, which may be an indication of temporal changes that occur during cancer progression being reflected in the systemic immune response. This possibility warrants further study.

Aberrant glycosylation has been a key feature in the acquisition and sustenance of hallmark characteristics that have been implicated in cancer development (44, 45). Furthermore the glycobiology of antibodies and their respective receptors have been suggested to be an important factor in mediated effector responses and therapeutic development and activity (46). Additionally, research has shown that 2,6 sialic acid expression is associated with chemoresistance in PDAC (47), albeit that data was based on altered sialylation of tumor antigens, not of tumor-associated autoantibodies as observed here. Whether these two observations of altered protein sialylation in PDAC are related functionally or not remains to be determined. Moreover, literature has shown the use of related bead-based immunoassays to profile the glycosylation patterns of pancreatic cancer serum proteins using SNA to distinguish between pancreatic cancer and chronic pancreatitis samples (36). Importantly, whilst we were able to determine the presence of specific glycan moieties on cancer antigen-specific serum autoantibodies using a microarray platform, we only focused on three possible glycan sites by using a panel of four lectins: SNA, LCA, RCA, and ECL, in order to respectively investigate sialylation (α2,6-Sal), fucosylation (α1,6-Fuc), and galactosylation (β1,4-Gal) of antigen-bound serum autoantibodies. Thus, improved coverage of all relevant glycan combinations would help us better characterize the role of altered glycosylation in PDAC autoantibodies. Indeed, more than 20 glycan variants are found on the IgG-Fc region in human sera (48). Furthermore, IgA effector functions and the associated glycosylation profiles are yet to be fully characterized or understood (46). Therefore, further research on the effect of glycosylation on antibody function is needed to establish their role in cancer progression.

Our study was limited by the available cohort size, particularly for the comparative site-of-disease *vs* serum analyses that were based on matched sera, tumor biopsy and normal adjacent biopsy samples from 8 patients, which is objectively a very small number. Albeit *post-hoc* power calculations show that this pilot, tissue-based study had >99% power to detect the observed changes in antibody class and subclass utilization, further research on a larger collection of matched tumor biopsy, normal adjacent biopsy and sera from PDAC patients will be required to validate these finding; this work is underway and will be reported elsewhere.

In addition, the deep quantitative analysis of antigen-specific isotype, subclass and glycosylation presented here is based on stated, pragmatic assumptions that might impact interpretation of the underlying biology and will benefit from verification via selective pre-purification and characterization of antigen-specific IgG and IgAs from a larger PDAC cohort in due course.

The combination of specific antigen reactivities in to the candidate biomarker panels reported here was based on statistically powered analysis of sera from a total of 89 patients (PDAC, n=30; disease controls, n=29; healthy controls, n=30). However, further validation in a larger, independent cohort will be required to confirm the clinical performance of these promising candidate serum autoantibody biomarkers of PDAC.

Overall, this study aimed to explore serum and tissue autoantibody reactivity against a panel of known cancer-testis and tumor-associated antigens using a high-throughput assay, revealing the presence of varying antibody subclass signatures and glycoforms that are associated with an immunosuppressive environment in PDAC tissue. Our data provides evidence of local autoantibody production at the site of disease, implying antigen presentation in the tumor microenvironment, albeit we are not yet able to identify the antigen presenting cell type. Nonetheless, our data suggests a significant perturbation of the immune environment during autoantibody production in PDAC tissue and potentially provides a simple means to track the effectiveness of future interventions that aim to restore an immunocompetent tumor microenvironment in PDAC.

## Data availability statement

The original contributions presented in the study are included in the article/**Supplementary Material**. Further inquiries can be directed to the corresponding author.

## Ethics statement

The studies involving humans were approved by University of Cape Town human research ethics committee (HREC 802/2020). The studies were conducted in accordance with the local legislation and institutional requirements. The participants provided their written informed consent to participate in this study.

## Author contributions

PM: Writing – original draft, Formal analysis, Writing – review & editing. MS: Supervision, Writing – review & editing. AN: Resources, Writing – review & editing. SB: Resources, Writing – review & editing. EJ: Conceptualization, Resources, Writing – review & editing. JB: Conceptualization, Supervision, Writing – review & editing, Writing – original draft.
