# Supplementary material for: Humoral immunoprofiling identifies novel biomarkers and an immune suppressive autoantibody phenotype at the site of disease in pancreatic ductal adenocarcinoma
Source: Front Oncol. 2024 Feb 21;14:1330419. doi: 10.3389/fonc.2024.1330419 (PMC10917065; doi:10.3389/fonc.2024.1330419)
Supplement: Supplementary file 2 [file Table_2.docx]

| Reagent | Stock concentration | Supplier | Catalogue number |
| --- | --- | --- | --- |
| α-hIgG | 2 mg/ml | ThermoFisher | A21145 |
| α-hIgA | 2.4mg/ml | ThermoFisher | 31140 |
| AF647-NHS ester | 10mg/ml | ThermoFisher | AF647-NHS ester |
| AF555-NHS ester | 10mg/ml | ThermoFisher | AF555-NHS ester |
| α-hIgA_1_ | 0.5mg/ml | Southern Biotech | 9130-01 |
| α-hIgA_2_ | 0.5mg/ml | Southern Biotech | 9140-01 |
| α-hIgG_1_ | 0.5mg/ml | Southern Biotech | 9054-01 |
| α-hIgG_2_ | 0.5mg/ml | Southern Biotech | 9060-01 |
| α-hIgG_3_ | 0.5mg/ml | Southern Biotech | 9210-01 |
| α-hIgG_4_ | 0.5mg/ml | Southern Biotech | 9200-01 |
| RCA | 10 mg/ml | Vector laboratories | L-1080-10 |
| SNA | 5 mg/ml | Vector laboratories | L-1300-5 |
| LCA | 10 mg/ml | Vector laboratories | L-1040-10 |
| ECL | 10 mg/ml | Vector laboratories | L-1140-10 |
| Biotin | 50 mM | Sigma® | B4501-1G |
| CaCl_2_ | 500 mM | Glentham | GK3739 |
| Glycerol | 50% | Sigma® | G7757-5L |
| HEPES | 2.5 M | Glentham | GM5581 |
| KCl | 1 M | Sigma® | P9333-1KG |
| Milk powder | Powder | Sigma® | 70166-500G |
| MgCl_2_ | 1000 mM | Glentham | GK5046 |
| MnCl_2_ | 100 mM | Glentham | GK2508 |
| PBS | 10x | Gibco | 70011-036 |
| Tris (pH 8.0) | 500 mM | Glentham | GP7166 |
| Triton X-100 | 100% | Sigma® | 93443-100ML |
| Tween 20 | 100 % | Glentham | GK2245 |
| Sodium bicarbonate | 0.1M | Glentham | GE8414 |

**Supplementary Table S2:** Reagents used for antibody and lectin derivatisation, and for microarray assays.
